# Evaluation of functioning in patients with schizophrenia and schizoaffective disorder

**DOI:** 10.1192/j.eurpsy.2026.12183

**Published:** 2026-07-31

**Authors:** E. Maaloul, R. Chakchouk, S. Ellouze, Y. Kammoun, M. Turki, J. Aloulou

**Affiliations:** Psychiatry B Department, CHU Hedi Chaker Sfax, Sfax, Tunisia

## Abstract

**Introduction:**

Functional recovery remains a major challenge in schizophrenia spectrum disorders. Even when clinical symptoms improve, many patients continue to experience significant impairments in autonomy, social relationships, and daily functioning.Examining the relationship between symptom burden and psychosocial functioning may help refine rehabilitation approaches.

**Objectives:**

This study aimed to evaluate functioning among patients with schizophrenia and schizoaffective disorder and to identify the main clinical and sociodemographic factors associated with functional impairment.

**Methods:**

A cross-sectional descriptive and analytical study was conducted among thirty-one patients diagnosed with schizophrenia or schizoaffective disorder. Symptom severity was evaluated using the Positive and Negative Syndrome Scale (PANSS). Psychosocial functioning was assessed with the Functioning Assessment Short Test (FAST).

**Results:**

The study sample consisted of 31 participants (48.4% men) with a mean age of 38.1 ± 9.3 years. The mean total FAST score was 22 ± 8.8, indicating a moderate level of functional impairment. Overall, 87.1% of participants exhibited impaired functioning in one or more domains. A significant association was observed between the total PANSS score and the total FAST score (p = 0.004). Among the FAST subdomains, autonomy (p = 0.013), interpersonal relationships (p = 0.042), and financial competence (p < 0.001) were significantly correlated with PANSS scores.

**Conclusions:**

In our study, greater symptom severity was associated with poorer psychosocial functioning, particularly in autonomy, interpersonal relationships, and financial competence. These results underline the close relationship between clinical symptomatology and functional outcome in schizophrenia and schizoaffective disorder, reinforcing the need for integrated, recovery-oriented interventions that address both symptom management and psychosocial rehabilitation.

**Disclosure of Interest:**

None Declared

